# Advanced Quantitative Microstructure Imaging in Autism: A Review of Methodology, Group Differences, and Associations With Developmental Outcomes

**DOI:** 10.1002/aur.70122

**Published:** 2025-10-06

**Authors:** Christy D. Yoon, Douglas C. Dean

**Affiliations:** ^1^ Waisman Center, University of Wisconsin‐Madison Madison Wisconsin USA; ^2^ Department of Medical Physics University of Wisconsin‐Madison Madison Wisconsin USA; ^3^ Department of Pediatrics University of Wisconsin‐Madison Madison Wisconsin USA

**Keywords:** advanced quantitative MRI, autism, CSD, DKI, microstructure, MTI, NODDI, relaxometry

## Abstract

Emerging evidence highlights widespread alterations in white matter microstructure in autism. Advances in magnetic resonance imaging (MRI) have enabled more precise examinations of these microstructural changes, leading to increased use of quantitative MRI techniques in autism research. This review summarizes the current landscape of these techniques, focusing on methodology, group differences, developmental associations, and regional variations. Following PRISMA guidelines, 34 studies published between 2006 and 2024 that employed advanced MRI techniques were reviewed. These included diffusion MRI signal representations (diffusion kurtosis imaging [DKI] and constrained spherical deconvolution [CSD]) and biophysical models (neurite orientation dispersion and density imaging [NODDI] and white matter tract integrity [WMTI]), as well as relaxometry and magnetization transfer imaging (MTI). CSD and NODDI were the most frequently used, while MTI was the least utilized, with notable variations in acquisition parameters and processing methods across the techniques. Findings suggest relatively consistent lower values of fixel‐based analysis measures (CSD) and neurite density index (NODDI) across major white matter regions, while findings from DKI, WMTI, and relaxometry varied. Measures from these techniques were associated with various developmental outcomes, including cognitive, emotional, and social behaviors. Limitations and implications are also discussed.

**Summary**

- Recent advances in magnetic resonance imaging (MRI) have facilitated detailed characterization and quantification of the brain's microstructural features.
- In particular, these techniques provide new insights into the ever‐evolving neurobiology of autism.
- This review summarizes the expanding body of research utilizing advanced MRI techniques to enhance our understanding of brain microstructure in autistic individuals, emphasizing notable variations in image acquisition and processing across all reviewed techniques.
- Despite these variations, relatively consistent group differences across major white matter regions and their relationship to various developmental outcomes have been observed, although some techniques have yielded mixed results.
- These findings highlight the need for greater consistency and long‐term studies in advanced brain imaging for autism research.

Autism is a complex neurodevelopmental condition characterized by differences in social communication, social interaction, and the presence of repetitive or restricted behaviors (American Psychiatric Association 2013). Importantly, autism is not a static condition; rather, it represents a lifelong developmental process defined by unique patterns of behavioral changes (Baghdadli et al. 2018; Georgiades et al. 2022; King and Bearman 2009; Magiati et al. 2014; Riglin et al. 2021; Sigman and McGovern 2005; Simonoff et al. 2020) and neural changes (Andrews et al. 2021; Courchesne et al. 2011; Hardan et al. 2009; Khundrakpam et al. 2017; Lange et al. 2015; Nunes et al. 2020; Prigge et al. 2021; Travers et al. 2015; Zielinski et al. 2014) that develop throughout childhood, adolescence, and adulthood.

There is substantial evidence suggesting that alterations in brain structure and function, such as changes in corpus callosum morphology, increased brain volume growth, and differences in brain connectivity, precede observable behavioral differences in children who develop autism (Dean et al. 2020; Emerson et al. 2017; Hazlett et al. 2017; Wolff et al. 2015; Wolff and Piven 2021). This implies that significant neural changes may occur before certain behavioral signs become evident, warranting ongoing and expanded investigation of neuroimaging techniques to track these gradual developmental shifts over time. Such imaging measures could provide important insights into how the brain responds to various interventions (Hegarty et al. 2019; Lei and Ventola 2017) and how it is influenced by factors contributing to quality of life, such as environmental contexts, mental health, and relationships (Backman et al. 2023; Bishop‐Fitzpatrick and Rubenstein 2019; Taylor and Mailick 2014; Woodman et al. 2014, 2015). Ultimately, neuroimaging measures sensitive to these incremental changes could improve long‐term outcomes for autistic individuals by enabling more precise monitoring of both neural and behavioral development.

Magnetic resonance imaging (MRI) is a powerful tool capable of non‐invasively assessing the brain, providing unique insights into the neurodevelopmental processes of autism. One promising approach to studying brain development in autism is diffusion tensor imaging (DTI; Alexander, Lee, Lazar, Boudos, et al. 2007; Alexander, Lee, Lazar, and Field 2007; Basser 1995; Basser et al. 1994). DTI is sensitive to the motion of water molecules and evaluates the microstructural properties of white matter (WM). Key DTI metrics include fractional anisotropy (FA), mean diffusivity (MD), axial diffusivity (AD), and radial diffusivity (RD). DTI has been extensively used to study WM microstructure in autism across different life stages, revealing altered and often distinct developmental trajectories in autistic individuals compared to neurotypical populations (Cheng et al. 2010; McLaughlin et al. 2018; Thompson et al. 2020; Travers et al. 2015; Wolff et al. 2012). Specifically, FA, one of the most commonly examined DTI metrics, tends to be elevated in younger autistic children (Faraji et al. 2023) but declines with age in older children, adolescents, and adults (Ameis and Catani 2015; Aoki et al. 2013; Travers et al. 2012). Conversely, RD is often reduced in younger autistic individuals (Faraji et al. 2023), while MD increases in older age groups (Ameis and Catani 2015; Aoki et al. 2013; Travers et al. 2012). These alterations in WM microstructure, often attributed to altered WM myelination in autism, have been most consistently observed in key tracts, including the corpus callosum, cingulum, superior longitudinal fasciculus, and uncinate fasciculus.

Despite its widespread use, DTI has notable limitations. For instance, DTI lacks the specificity needed to distinguish between different types of microstructural changes (Andica et al. 2020; Jones et al. 2013), such as changes to fiber organization, axonal damage, or myelin abnormalities. Additionally, DTI is limited in its ability to resolve multiple fiber orientations in regions with crossing fibers, which can result in less reliable estimates of WM integrity (Jeurissen et al. 2013). Furthermore, DTI's assumption of Gaussian water diffusion (Wheeler‐Kingshott and Cercignani 2009) may not hold in more complex tissue environments, and partial volume effects from cerebrospinal fluid can further decrease its reliability (Alexander et al. 2001), particularly when applied to gray matter (GM) analysis. To address these limitations, research has increasingly focused on more advanced quantitative MRI techniques that offer greater precision in measuring brain microstructure.

## 1 Overview of Advanced Quantitative Microstructure Imaging Techniques

Among advanced quantitative MRI techniques, diffusion MRI has emerged as a prominent method, with techniques broadly classified into signal representations (or statistical models) and biophysical models (Jelescu et al. 2020). Complementary techniques, such as relaxometry and magnetization transfer imaging (MTI), offer additional insights into microstructural properties by probing distinct tissue characteristics in conjunction with diffusion MRI.

### 1.1 Diffusion MRI Signal Representations

Diffusion MRI signal representations, including diffusion kurtosis imaging (DKI) and constrained spherical deconvolution (CSD), directly characterize diffusion signals without relying on assumptions about the underlying tissue architecture (Jelescu et al. 2020; Pierpaoli et al. 1996).

*DKI* extends conventional DTI by modeling water diffusion in a non‐Gaussian manner (Hui et al. 2008; Jensen et al. 2005; Steven et al. 2014). To achieve this, DKI protocols require the acquisition of multi‐shell diffusion‐weighted images with at least two non‐zero *b*‐values and a minimum of 15 unique diffusion gradient directions (De Santis et al. 2011; Veraart et al. 2011). This model is more suitable for complex brain tissues where diffusion deviates from Gaussian assumptions. DKI provides additional scalar metrics, including mean kurtosis (MK), axial kurtosis (AK), radial kurtosis (RK), and fractional anisotropy of kurtosis (FAK; Hansen and Jespersen 2016; Jensen and Helpern 2010). MK indicates the average degree of diffusion restriction across all directions, with higher MK values suggesting increased tissue complexity or microstructural barriers. AK reflects kurtosis along the axonal direction and is suggested to be affected by intracellular structures, whereas RK reflects kurtosis along perpendicular to axons and is suggested to be influenced by cellular membranes and myelin sheaths (Steven et al. 2014). FAK quantifies the extent to which kurtosis varies with direction, analogous to FA in DTI; higher FAK values indicate greater directional dependence of microstructural complexity, highlighting tissues where kurtosis exhibits strong anisotropy, such as organized WM tracts (Hansen and Jespersen 2016). Rapid increases in these metrics are typically observed during infancy and early childhood (Paydar et al. 2014; Shi et al. 2019), potentially reflecting intensive processes such as myelination and cellular organization. Beyond this developmental period, maturation proceeds in a more gradual and region‐specific manner throughout later childhood into early adolescence, with ongoing changes notable in certain association regions (Shi et al. 2019), followed by degenerative processes in late adulthood (Benitez et al. 2018).

*CSD* (Dhollander and Connelly 2016; Jeurissen et al. 2014; Tournier et al. 2004, 2007) estimates the WM fiber orientation distribution at each voxel by using high‐angular‐resolution diffusion imaging with at least one non‐zero *b*‐value. This enables CSD to resolve crossing fibers and overcome a limitation of conventional DTI, which assumes a single dominant diffusion direction per voxel. By estimating multiple fiber orientations, CSD facilitates a more accurate mapping of complex WM pathways, particularly in regions where fibers intersect. This advantage is especially important in fiber tractography, where CSD fiber orientation distributions provide a more robust representation of the underlying WM fiber orientations. Techniques such as fixel‐based analysis (Raffelt et al. 2015, 2017) enable the quantification of distinct fiber populations within a voxel. For example, fiber density (FD) reflects the intra‐axonal volume fraction aligned with a given fiber orientation and serves as a proxy for axonal density; fiber cross‐section (FC) captures macroscopic differences in the cross‐sectional area of fiber bundles, derived from spatial deformations during image registration, and is sensitive to tract expansion or compression; and the combined metric, FD and FC (FDC), is computed as the product of FD and FC, providing an integrated measure of both micro‐ and macrostructural properties of WM. Higher values of FD, FC, and FDC may indicate increased axonal content, larger bundle morphology, and greater total fiber capacity, respectively. In typical development, these measures have been generally shown to increase with age but at different rates across the brain, reflecting progressive and regionally specific axonal growth and WM expansion during development. For instance, Genc et al. (2018) observed a longitudinal increase in FD, FC, and FDC across late childhood, especially in commissural and association fibers. DiPiero et al. (2022) further reviewed consistent age‐related increases in CSD‐derived measures during early development, supporting their sensitivity to dynamic WM maturation.

### 1.2 Diffusion MRI Biophysical Models

Unlike signal representations, biophysical models such as the composite hindered and restricted model of diffusion (CHARMED), neurite orientation dispersion and density imaging (NODDI), and white matter tract integrity (WMTI) aim to model the microstructural geometry of tissues and offer metrics that are more biologically meaningful and readily interpretable (Jelescu et al. 2020; Jelescu and Budde 2017).

*CHARMED* (Assaf et al. 2004, 2008; Assaf and Basser 2005) is designed to distinguish between hindered diffusion in the extracellular space, characterized by Gaussian decay, and restricted diffusion within the intracellular space, characterized by non‐Gaussian decay. CHARMED requires the acquisition of multi‐shell diffusion‐weighted images with at least two non‐zero *b*‐values and between 30 and 60 diffusion gradient directions to reliably estimate its parameters (Nazeri et al. 2020). This is achieved by modeling WM as a composite of parallel cylindrical axons with a gamma distribution of radii. CHARMED estimates parameters such as the fraction of the signal from restricted diffusion, which is presumed to reflect the composition of the intra‐axonal compartment (Assaf and Basser 2005). Higher values of this restricted component are generally associated with increased axonal density or intra‐axonal content (Assaf et al. 2008), while a higher hindered component reflects diffusivity, potentially indicating reduced structural complexity or fewer barriers in the extracellular space (e.g., due to axonal loss; Assaf and Basser 2005). In typical development, CHARMED‐derived estimates of the intra‐axonal signal fraction differentiate between early‐ and late‐maturing WM regions, reflecting WM maturation and the expansion of the restricted compartment (DiPiero et al. 2022; Kunz et al. 2014).

*NODDI* (Zhang et al. 2012) distinguishes among intracellular, extracellular, and isotropic water compartments. Similar to CHARMED, NODDI requires a multi‐shell diffusion protocol with at least two non‐zero *b*‐values and 30 to 60 diffusion gradient directions to reliably estimate its parameters (Nazeri et al. 2020; Zhang et al. 2012): the intracellular volume fraction, known as the neurite density index (NDI), which reflects the volume fraction of tissue occupied by neurites; the orientation dispersion index (ODI), which quantifies the angular spread of neurite orientations; and the isotropic volume fraction (FISO), representing the fraction of free water. Higher NDI values indicate a greater number of axons and dendrites (i.e., neurites), often associated with increased microstructural complexity or maturation; elevated ODI values reflect more dispersed neurite orientations, characteristic of cortical or complex fiber regions; and increased FISO suggests higher free water content, often due to cerebrospinal fluid, edema, atrophy, or inflammation. During typical brain development, NDI increases from infancy through adolescence, reflecting neurite growth and myelination primarily in WM (Mah et al. 2017; Zhao et al. 2021). ODI is also observed to increase with age in GM from infancy through adolescence, reflecting greater neurite orientation complexity in GM and more subtle refinements in WM organization (Zhao et al. 2021).

*WMTI* is a biophysical model based on DKI that characterizes WM as two compartments: the intra‐axonal and extra‐axonal spaces (Fieremans et al. 2010, 2011). By integrating DKI measurements within this two‐compartment framework, WMTI estimates four parameters (Fieremans et al. 2013; Jelescu et al. 2015): the axonal water fraction (*f*_*axon*_), indicating axonal density; intra‐axonal diffusivity (*D*_*axon*_), reflecting axonal integrity; extra‐axonal axial (*AD*_*extra*_) and radial diffusivities (*RD*_*extra*_), capturing changes in the extra‐axonal environment, with *RD*_*extra*_ being particularly sensitive to myelin integrity; and extra‐axonal tortuosity, reflecting the extent to which myelinated axons restrict radial diffusion, with higher values suggesting more intact and densely packed fibers. During typical development, especially within the first 3 years of healthy brain development, a non‐linear increase in *f*_*axon*_ and in extra‐axonal tortuosity has been observed (Jelescu et al. 2015).

### 1.3 Relaxometry

Relaxometry is a quantitative MRI technique that measures relaxation times (*T*_*1*_, *T*_*2*_, *T*_*2*_ *'*; Deoni 2010; Deoni and Dean 2021) and offers an alternative to DTI for assessing microstructural changes in the brain in vivo. It provides detailed insights into molecular interactions, energy exchanges, and the biochemical environment of tissues by examining the behavior of water protons in various contexts (Moody et al. 2022). *T*_*1*_ represents energy transfer between water protons and surrounding macromolecules, whereas *T*_*2*_ measures the dephasing of water protons due to interactions with local tissue structures (Deoni 2010; Deoni and Dean 2021). Both *T*_*1*_ and *T*_*2*_ are sensitive indicators of the presence of myelin and cellular structures critical for maintaining WM integrity (Dean et al. 2017; Deoni and Dean 2021; Leppert et al. 2009). In addition to these metrics, myelin water fraction (MWF), derived through multi‐component relaxometry (Dean et al. 2014, 2017; Deoni et al. 2008, 2015; MacKay et al. 2006), offers a more specific estimate of myelin content by quantifying the proportion of water trapped between myelin layers. *T*_*2*_ *'*, a related metric that enhances sensitivity to magnetic field inhomogeneities, captures subtle tissue variations that may go undetected by conventional imaging. Typically, higher *T*_*1*_ and *T*_*2*_ values indicate reduced tissue organization or lower macromolecular content. Conversely, higher MWF values indicate greater myelin content. *T*_*2*_ *'*, which is sensitive to magnetic susceptibility, decreases with iron accumulation or vascular pathology. Normatively, *T*_*1*_ and *T*_*2*_ values decrease during development, a change associated with progressive myelination and other structural maturation (Deoni et al. 2012; Eminian et al. 2018). Similarly, MWF tends to increase throughout infancy and early childhood, reflecting rapid myelin accumulation (Dean et al. 2014).

### 1.4 Magnetization Transfer Imaging (MTI)

MTI is a quantitative MRI technique that examines brain microstructure by measuring the exchange of magnetization between free water protons and macromolecular protons (Graham and Henkelman 1999; Sled 2018; van Buchem et al. 2001; Wolff and Balaban 1989). The primary metric derived from MTI is the magnetization transfer ratio (MTR), which indicates the efficiency of magnetization transfer and serves as an indirect marker of macromolecular content (Anik et al. 2007; Berry et al. 1999; Fjær et al. 2013; Kabani et al. 2002). Other metrics, including the peak location and height of the MTR histogram, contribute to understanding the overall integrity and distribution of tissue properties. Higher MTR values typically signify greater macromolecular content, often linked to myelin content. In spectral analyses, a lower peak height or shifted peak position may indicate diffuse or heterogeneous tissue damage, such as demyelination or inflammation. MTI is particularly sensitive to myelin integrity (Brouwer et al. 2010; Filippi and Rocca 2004; Mandl et al. 2010), making it a more reliable indicator of myelin content compared to DTI. During typical brain development, MTR has been observed to increase nonlinearly during early childhood, especially in the first 2 years of life (Zhang et al. 2016), indicative of rapid myelination in WM regions, while showing no significant change during late childhood (Moura et al. 2016), suggesting a plateau phase, and remaining relatively stable throughout adulthood (Mehta et al. 1995). See Table S1 for a summary of biological features measured by the parameters of each MRI technique.

## 2 Current Review

To the best of our knowledge, while several reviews of DTI in autism exist (e.g., Ameis and Catani 2015; Faraji et al. 2023; Travers et al. 2012), only one review to date focuses on the application of advanced quantitative microstructure imaging techniques (DiPiero et al. 2022). DiPiero et al. review provides a comprehensive synthesis of the use, findings, and limitations of advanced diffusion MRI methods, including CHARMED, CSD, DKI, and NODDI, primarily in pediatric research. Their findings highlight the superior sensitivity of these techniques in identifying microstructural changes and suggest distinct developmental patterns in both GM and WM. While this review delivers valuable insights into early brain development, it is essential to expand its scope to encompass autism across the lifespan and to explore additional advanced quantitative microstructure imaging techniques, such as relaxometry and MTI, that reveal key and complementary characteristics of tissue properties. Thus, this review aims to present an overview of the current state of advanced quantitative microstructural imaging techniques in autism research to deepen our understanding of the utility, sensitivity, and existing gaps in our knowledge of the neurobiology of autism across the lifespan. Specifically, we synthesize: (a) methodological aspects, including imaging acquisition protocols and processing methods; (b) the sensitivity of these imaging metrics in characterizing group differences and their associations with developmental outcomes, including behavioral, cognitive, and emotional domains; and (c) similarities and differences among the brain regions across the techniques.

## 3 Methods

**FIGURE 1:**
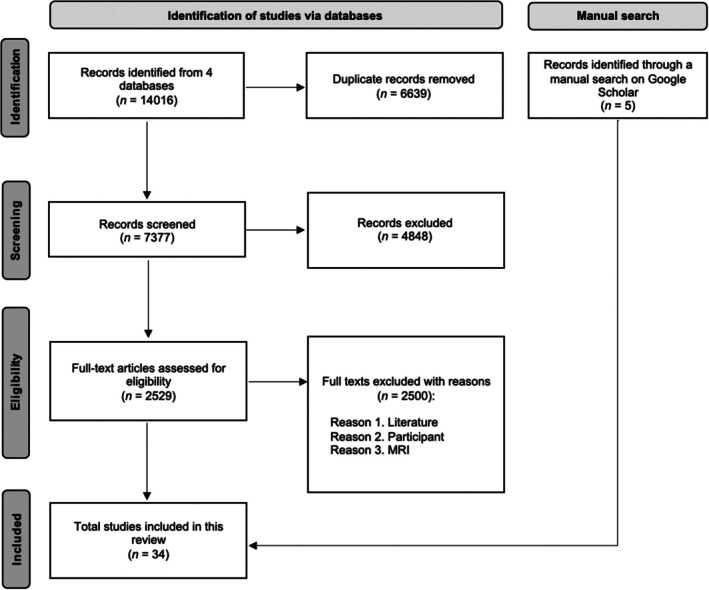
PRISMA flow chart.

To identify studies that utilized advanced quantitative microstructure imaging techniques in autism, we adhered to the Preferred Reporting Items for Systematic Reviews and Meta‐Analyses (PRISMA; Page et al. 2021) guidelines for conducting systematic reviews (Figure 1).

### 3.1 Search Process

The initial search was performed using four electronic databases in May 2024: MEDLINE, PsycArticles, PsycInfo, and PubMed. The key search terms pertained to autism (“autism” OR “*ASD*” OR “autism spectrum disorder” OR “autis*”) and MRI (“magnetic resonance imaging” OR “*MRI*”). The initial search yielded 14,016 articles. After removing 6639 duplicates, 7377 articles remained for screening.

### 3.2 Screening Process

The inclusion criteria for this review were as follows: each study (a) was published in English in a peer‐reviewed journal; (b) contained empirical data; (c) included all or at least one group of participants diagnosed with autism; and (d) applied advanced quantitative microstructure imaging in vivo. To ensure a more comprehensive synthesis of the available literature, we did not restrict the publication period or participant age. However, we excluded studies where participants with autism were part of a non‐homogeneous group, as well as studies involving animal models of autism. We also excluded studies that utilized CSD to estimate fiber orientation distribution but did not derive fixel‐based metrics. Lastly, we excluded studies that exclusively used DTI or solely focused on brain morphometry, as reviews of these studies are available (Ameis and Catani 2015; Brambilla 2003; Faraji et al. 2023; Travers et al. 2012).

### 3.3 Study Selection Process

The inclusion criteria were applied to the titles and abstracts of 7377 studies, resulting in the exclusion of 4848 articles. Consequently, the remaining 2529 articles underwent full‐text assessment, which led to the exclusion of an additional 2500, leaving 29 articles that met the inclusion criteria. Furthermore, a manual search on Google Scholar in August 2024 yielded five articles that also met the inclusion criteria. As a result, 34 articles were included in this review.

### 3.4 Data Extraction

For each of the 34 studies, the following variables were extracted: (a) participant characteristics (sample size, age, sex); (b) the advanced quantitative microstructure imaging technique used; (c) details of the MRI acquisition protocol (magnetic field strength, MRI manufacturer, matrix size, field of view [FOV], image resolution, repetition time [TR], echo time [TE], *b*‐value, diffusion direction); specifically, matrix size, FOV, or in‐plane resolution was calculated (*FOV = matrix size × in‐plane resolution*) when not reported; (d) tools used to process data; (e) derived metrics; and (f) key findings on group differences and associations with developmental outcomes. For the last variable, we extracted results that were corrected for multiple comparisons when both uncorrected and corrected results were available and excluded results representing aggregated samples of autism and control groups.

## 4 Results

**FIGURE 2:**
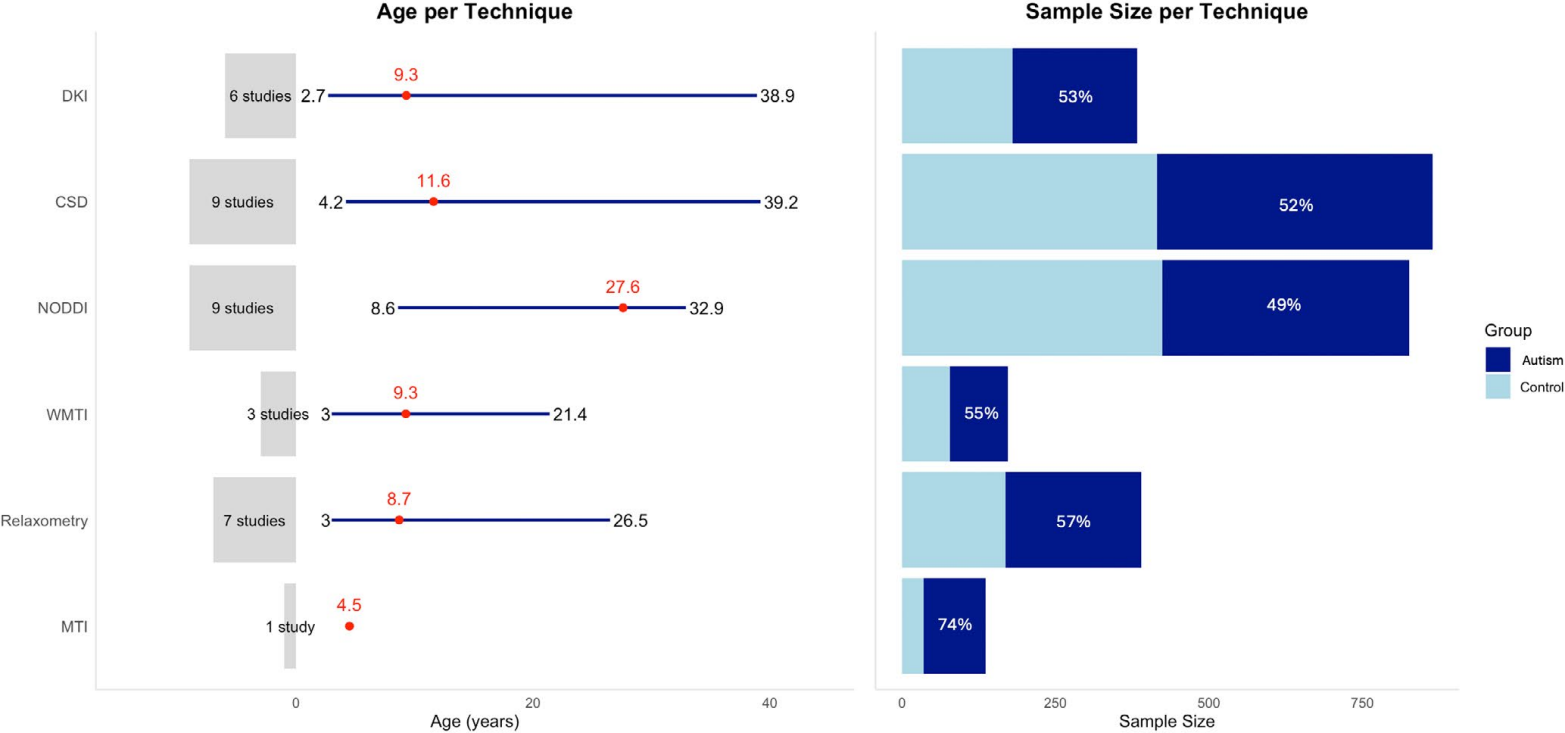
Age of autistic participants (mean age and range of mean age in years) and sample size by technique. *Note*: The mean age range does not apply to MTI, as there is only one study that utilized MTI. One study (Shen et al. 2024) is included in both DKI and WMTI.

This review encompasses 34 studies published between 2006 and 2024 that utilized advanced quantitative microstructure imaging techniques: DKI (*n* = 6), CSD (*n* = 9), NODDI (*n* = 9), WMTI (*n* = 3), relaxometry (*n* = 7), and MTI (*n* = 1). Collectively, it includes 2665 participants (*N*_*autism*_ = 1408, *M*_*age*_ = 2.8–39.8 years, 79% male; *N*_*control*_ = 1257, *M*_*age*_ = 2.8–40 years, 75% male). The results are organized by methodological features, group differences, and associations with developmental outcomes for each technique employed, followed by a synthesis of similarities and differences among the brain regions across these techniques. See Table 1 for the characteristics of the included studies and Figure 2 for an illustration of these characteristics by technique.

**TABLE 1:** Reviewed study information (*n* = 34), including participant characteristics and key findings, organized by diffusion MRI signal representations (DKI, CSD), diffusion MRI biophysical models (NODDI, WMTI), relaxometry, and MTI.

Authors (year)	Autism group		Control group		Key findings
	*N* (male%)	Mean age in years (SD)	*N* (male%)	Mean age in years (SD)	
Diffusion MRI signal representations					
Diffusion kurtosis imaging (DKI)					
Hattori et al. (2019)	15 (67%)	38.9 (7.9)	15 (47%)	39.8 (7.9)	Lower AK (in the splenium and body of the corpus callosum) in the autism group relative to the NT group. No group differences in MK and RK. Negative correlations between AK (predominantly in WM areas related to social‐emotional processing) and AQ or SQ in the autism group.
He et al. (2023)	32 (72%)	2.8 (0.8)	27 (70%)	2.5 (1.0)	Lower AK and FAK of multi‐fibers (such as the bilateral superior longitudinal fasciculus, corticospinal tract, and anterior thalamic radiation) in the autism group relative to the NT group. No group differences in MK and RK.
McKenna et al. (2020)	16 (100%)	21.4 (2.4)	17 (100%)	21.7 (2.1)	Decreased MK and RK (in the frontal and temporal lobes and several sub‐lobar regions) in the autism group relative to the NT group. Negative association between MK (in the left entorhinal cortex) and ADI‐R Repetitive and Restrictive Behaviors, and between MK (in the right parietal lobe) and ADI‐R Social Interaction in the autism group.
Nagai et al. (2024)	18 (72%)	29.1 (7.0)	17 (94%)	32.7 (8.5)	Increased AK and MK in WM tracts (right anterior corona radiata, forceps minor, and right superior longitudinal fasciculus) in the autism group relative to the NT group. No group differences in RK. A negative correlation between MK (right superior longitudinal fasciculus, splenium of the corpus callosum, right corticospinal tract, right superior corona radiata, right posterior corona radiata, right retrolenticular part of the internal capsule, and right posterior thalamic radiation) and EQ, and between RK (in the body and splenium of the corpus callosum) and AQ‐Imagination in the autism group.
Shen et al. (2024)	62 (73%)	3 (n/r)	44 (61%)	3.0 (n/r)	Increased AK, MK, and FAK (in the fornix, corpus callosum, bilateral internal capsule anterior and posterior limbs, and bilateral cerebral peduncle) in the autism group relative to the NT group.
Tang et al. (2022)	60 (53%)	2.7 (n/r)	60 (52%)	2.8 (n/r)	Lower AK, RK, and AK (in the frontal and temporal lobes, hippocampus, caudate nucleus, substantia nigra, and red nucleus) and FAK (in the frontal and temporal lobes and hippocampus) in the autism group relative to the NT group.
Constrained spherical deconvolution (CSD)					
Dimond et al. (2019)	25 (84%)	16.8 (2.3)	27 (78%)	16.9 (2.1)	Reduced FD in several major WM tracts (corpus callosum, bilateral inferior frontal‐occipital fasciculus, right arcuate fasciculus, right uncinate fasciculus) in the autism group relative to the NT group. A negative association between FD (in the splenium of the corpus callosum) and SRS‐2 in the autism group.
Dong et al. (2023)	26 (100%)	39.2 (16.2)	26 (100%)	40 (15.6)	No group differences in FD, FC, or FDC.
Guerrero‐Gonzalez et al. (2022)	56 (79%)	8.5 (1.3)	72 (65%)	8.3 (1.4)	A methodology paper aimed to improve the quality of TiDi Fused post‐processing method in autistic children.
Kirkovski et al. (2020)	25 (48%)	30.6 (9.1)	24 (50%)	30.3 (10.1)	Reduced FDC (in the corpus callosum) in autistic females relative to NT females. No differences in FD or FC across WM between female and male subgroups, and no differences in FDC across WM between autism and NT groups, or between male subgroups.
Kirkovski et al. (2023)	54 (85%)	Site 1: 11.2 (7.5); site 2: 13.9 (3.2); site 3: 17.1 (3.6)	50 (94%)	Site 1: 10.0 (4.4); site 2: 13.9 (2.9); site 3: 16.6 (3.0)	Reduced log FC and FDC (around the midbody, isthmus, and splenium regions of the corpus callosum) in the younger autistic adolescents relative to the NT group; and reduced FD (in a small region of the splenium) and FDC (at the midbody) in older autistic adolescents relative to the NT group. No group differences in young adult cohorts.
Newman et al. (2024)	148 (53%)	4.2 (2.9)	124 (50%)	4.5 (2.9)	Widespread increases in extracellular water (in the cortex), decreases in aggregate g‐ratio (throughout the cortex, subcortex, and WM skeleton), and decreases in aggregate conduction velocity (throughout the cortex, subcortex, and WM skeleton) in the autism group relative to NT group. A positive association between extracellular water signal fraction and SCQ in 55 ROIs, a negative association between aggregate g‐ratio and SCQ in 12 ROIs, and a negative association between aggregate conduction velocity and SCQ in 139 ROIs (including both composite cortical and WM ROIs) in the autism group.
Ni et al. (2023)	22 (82%)	13.0 (3.2)	Autism sham: 27 (85%)	12.9 (2.8)	No differences in FD, FC, or FDC across WM between treatment groups at baseline, and within groups post‐4 weeks or 8 weeks treatment. No associations between baseline FD, FC, or FDC across WM and changes in SRS from baseline to week 4 treatment.
Yeh et al. (2022)	65 (91%)	16.6 (5.9)	38 (79%)	17.3 (5.7)	Smaller FD (in the anterior and posterior corpus callosum and the left cerebellum Crus I) and FDC (in the right cerebellum Crus II) in the whole autism group, and smaller FD (in the anterior and posterior) and FDC (in the corpus callosum and right Crus II) in the autism subgroup with intellectual disability, relative to the NT group. No differences between the NT and the autism subgroup without intellectual disability, or between the autism subgroups. A negative correlation between FD or FDC (of the dorsolateral prefrontal cortex) and BRIEF‐GEC, and between WM FC (in the right cerebellum Crus I) and ADOS‐2 CSS in the autism group. No correlations with SRS or VABS in the autism group.
Yeh et al. (2024)	28 (93%)	13.0 (3.4)	Autism sham: 27 (89%)	13.1 (2.8)	Greater baseline FD (at the body and isthmus of the corpus callosum) in the active theta burst stimulation group relative to the sham group. No baseline group differences in FC and FDC across WM, and no post‐treatment group differences in FD, FC, or FDC across WM. No association between baseline FD, FC or FDC across WM and autism symptom changes from baseline to week 8 in the active theta burst stimulation group.
Diffusion MRI biophysical models					
Neurite orientation dispersion and density imaging (NODDI)					
Andica et al. (2021)	26 (73%)	32.9 (9.2)	25 (68%)	34.4 (9.0)	Lower NDI and higher FISO (mainly in commissural and long‐range association tracts) in the autism group relative to the NT group. No association between NDI (in the left anterior thalamic radiation, superior longitudinal fasciculus, or uncinate fasciculus) and AQ in the autism group.
Arai et al. (2023)	22 (64%)	32.6 (9.6)	26 (65%)	34.4 (8.3)	Lower NDI (in the left prefrontal cortex) in the autism group relative to the NT group. No group differences in ODI and FISO. A positive association between NDI (in the left rostral middle frontal, superior frontal, and left frontal pole) and EQ in the autism group.
DiPiero, Cordash, et al. (2023)	78 (100%)	26.7 (7.3)	81 (100%)	27.0 (6.8)	Lower WM NDI and higher GM and WM ODI across widespread WM tracts in the autism group relative to the NT group. No group differences in GM NDI. No associations with ADOS‐CSS or SRS in the autism group.
DiPiero, Surgent, et al. (2023)	70 (100%)	12.1 (3.8)	83 (100%)	12.7 (4.7)	Lower NDI (across frontal, temporal, and occipital regions of the right hemisphere) in the autism group relative to the NT group. No group differences in ODI. A negative association between ODI (in areas largely localized to the right hemisphere) and ADOS‐2 CSS in the autism group.
Kitamura et al. (2023b)	41 (73%)	28.2 (6.5)	39 (72%)	27.5 (5.4)	No group differences in NDI (in supplementary motor area, superior frontal gyrus, supramarginal gyrus, superior temporal gyrus, or precuneus). A positive association between NDI (in the bilateral supplementary motor area, right superior frontal, left supramarginal, right superior temporal gyrus, and right precuneus) and posttraumatic stress disorder symptom severity, and between NDI (in the right superior frontal and left supramarginal gyrus and right precuneus) and adverse childhood experience severity, as measured by CATS, in the autism group.
Kitamura et al. (2023a)	45 (76%)	28.5 (6.7)	43 (72%)	27.3 (5.2)	Higher NDI (in the right superior temporal gyrus) in the autism group with high sensory over‐responsivity relative to the autism group with the low sensory over‐responsivity and NT group. No differences in NDI (in the right superior temporal gyrus) between the autism group with low sensory over‐responsivity and the NT group. A positive association between NDI (in the right superior temporal gyrus) and sensory over‐responsivity severity, as measured by AASP‐SOR, and adverse childhood experiences, as measured by CATS, in the autism group. No associations between ODI and AASP‐SOR or CATS in the autism group.
Matsuoka et al. (2020)	28 (86%)	28.9 (6.0)	29 (83%)	27.3 (4.9)	Higher ODI (in the left occipital gyrus) in the autism group relative to the NT group. No group differences in AK in either lobar or sub‐lobar regions. Positive associations between ODI (in the left occipital gyrus) and low registration and passive behavioral response characteristics of the visual quadrants of the AASP in the autism group.
Travers et al. (2024)	74 (80%)	8.6 (n/r)	71 (66%)	8.2 (n/r)	A positive association between a summary measure reflecting decreased FA and AD and increased RD and ODI (of the bilateral parvicellular reticular formation alpha cluster) and a summary measure of SRS‐2, SCQ, and RBS‐R in the autism group.
Yasuno et al. (2020)	18 (89%)	28.9 (9.2)	27 (93%)	25.5 (2.7)	Smaller NDI and ODI (ventral occipital complex region encompassing the fusiform gyrus, inferior parietal region, and forceps major of the corpus callosum) in autistic individuals with high IDCS relative to the NT group. No differences in NDI and ODI (across the same regions) between the autism and NT groups. Negative correlations between NDI or ODI (in clusters including the ventral occipital complex region, superior temporal/parietal association areas, and forceps major of the corpus callosum) and difficulties in facial emotional recognition in horizontal slit‐viewing, as indexed by IDCS, in the autism group.
White matter tract integrity (WMTI)					
Lazar et al. (2014)	16 (100%)	21.4 (2.4)	17 (100%)	21.7 (2.1)	Decreased *f*_*axon*_ in most of the major WM tracts (including the corpus callosum, cortico‐spinal tracts, and superior longitudinal, inferior longitudinal and inferior fronto‐occipital fasciculi), decreased *D*_*axon*_ (in some of the WM regions), and decreased *AD*_*extra*_ (in localized area of the prefrontal corpus callosum) in the autism group relative to the NT group. No groups differences in *RD*_*extra*_. A positive correlation between WAIS‐III‐Digit Symbol‐Coding and *f*_*axon*_ and *D*_*axon*_ (of the left inferior longitudinal and left fronto‐occipital fasciculi) in the autism group.
Shen et al. (2024)	62 (73%)	3 (n/r)	44 (61%)	3.0 (n/r)	Increases in *f*_*axon*_, *AD*_*extra*_, tortuosity, and *D*_*axon*_ (primarily in the corpus callosum and fornix) in the autism group relative to the NT group. No group differences in *RD*_*extra*_.
Sui et al. (2018)	16 (100%)	21.4 (2.4)	17 (100%)	21.7 (2.1)	Decreased callosal *f*_*axon*_ and *D*_*axon*_ (particularly in the midbody, isthmus, and splenium of the corpus callosum) in the autism group relative to the NT group. No group differences in *AD*_*extra*_ and *RD*_*extra*_. No association between WMTI metrics and WAIS‐III‐ Digit Symbol‐Coding and Symbol Search (in the mid and posterior segments) in the autism group.
Relaxometry					
Deoni et al. (2015)	14 (100%)	24.2 (1.21)	14 (100%)	27.9 (1.6)	Increased *T*_*1*_ (bilaterally within the cerebellum, thalamus, internal capsule, and in right temporal and occipital WM) and reduced MWF (bilaterally within the cerebellum, thalamus, internal capsule, caudate nuclei, temporal and occipital WM, SMA and pre‐SMA, cingulum, and right frontal WM) in the autism group relative to the NT group. No group differences in *T*_*2*_. A negative correlation between MWF (bilaterally in the thalamus, caudate, and in the right frontal lobe and external capsule) and total ADOS score, between MWF (bilaterally in the thalamus, right temporal and frontal lobes, and cingulum) and ADOS Social Interaction, and between MWF (in the right cerebellum, occipital lobe and superior corona radiata) and AQ in the autism group. No correlations between *T*_*1*_ or *T*_*2*_ and ADI, ADOS, or AQ in the autism group.
Fischi‐Gomez et al. (2021)	16 (81%)	26.5 (2.5)	12 (83%)	26.8 (2.3)	No group differences in *T*_*1*_ (in amygdala and orbitofrontal cortex).
Hasan et al. (2013)	10 (90%)	10.9 (2.4)	10 (90%)	11.4 (2.0)	No group differences in *T*_*2*_ (in whole brain GM and WM).
Hendry et al. (2006)	19 (100%)	9.2 (3.0)	20 (100%)	10.7 (2.8)	Longer mean cerebral WM *T*_*2*_ in the autism group relative to the NT group. No group differences in mean cerebral GM *T*_*2*_, and GM or WM *T*_*2*_ *′*.
Li et al. (2023)	34 (82%)	3 (n/r)	GDD: 17 (76%)	2.7 (0.6)	Shortened *T*_*1*_ (of the genu and splenium of the corpus callosum and right thalamus) and *T*_*2*_ (of the genu of the corpus callosum, left parietal WM, and bilateral thalamus) in the autism group relative to the GDD group. Positive correlations between *T*_*2*_ (of the left parietal WM and left thalamus) and DQ2 and DQ5, and between *T*_*2*_ (of the genu of corpus callosum) and DQ5 in the autism group. No correlations with CARS in the autism group.
Petropoulos et al. (2006)	60 (80%)	3.5 (0.91)	DD: 16 (44%); NT: 10 (80%)	3.7 (0.8)	In the autism group, lower *T*_*2*_ (in cortical GM and WM) relative to the DD group, and prolonged *T*_*2*_ (in cortical GM) relative to the NT group. No differences in WM *T*_*2*_ between autism and NT groups. No correlation between *T*_*2*_ (GM and WM) and IQ measures in the autism group.
Surgent et al. (2024)	68 (81%)	8.4 (1.36)	70 (66%)	8.3 (1.4)	A positive association between *R*_*1*_ (inverse of *T*_*1*_) and grip strength, as measured by the Jamar hand dynamometer, in the autism group and in autistic children with elevated ADHD. A negative association between *R*_*1*_ and grip strength in autistic children with reduced ADHD features.
Magnetization transfer imaging (MTI)					
Gozzi et al. (2012)	101 (81%)	4.5 (1.4)	35 (66%)	4.0 (1.7)	Higher mean MTR and MTR histogram peak height and location (in the corpus callosum) in the autism group relative to the NT group. No correlations with nonverbal DQ in the autism group.

*Note*: A few studies that provided mean ages in months have been converted to years for uniformity across the studies. Only post‐correction results are reported for key findings on group differences and associations. The neurotypical group reflects control groups reported as typically developing or healthy controls for consistency in terminology. Shen et al. (2024) is included in both DKI and WMTI categories because it employed both techniques. The gray shading indicates the MRI techniques reviewed in this study, along with the corresponding studies.

Abbreviations: AASP‐SOR = adolescent/adult sensory profile‐sensory over‐responsivity; AD = axial diffusivity; *AD*_*extra*_ = axial extra‐axonal diffusivity; ADHD = attention‐deficit/hyperactivity disorder; ADI‐*R* = autism diagnostic interview‐revised; ADOS‐2 CSS = autism diagnostic observation schedule‐2 calibrated severity scores; AK = axial kurtosis; AQ = autism spectrum quotient; BRIEF‐GEC = behavior rating inventory of executive function‐global executive composite; CARS = Childhood Autism Rating Scale; CATS = Child Abuse Trauma Scale; *D*_*axon*_ = intra‐axonal diffusivity; DD = developmental delay; DQ = Gesell Developmental Scale; DQ2 = Gesell Developmental Scale of Gross Motor; DQ5 = Gesell Developmental Scale of Personal‐Social Behavior; EQ = empathizing quotient; FA = fractional anisotropy; FAK = fractional anisotropy of kurtosis; *f*_*axon*_ = axonal water fraction; FC = fiber cross‐section; FD = fiber density; FDC = fiber combined measure; FISO = isotropic volume fraction; GBSS = gray matter‐based spatial statistics; GDD = global developmental delay; GM = gray matter; IDCS = index of difference in correctness between vertical and horizontal slits; MK = mean kurtosis; MTR = magnetization transfer ratio; MWF = myelin water fraction; NDI = neurite density index; NT = neurotypical; ODI = neurite orientation dispersion index; *R*_*1*_ = longitudinal relaxation rate; RBS‐*R* = Repetitive Behavior Scale‐Revised; RD = radial diffusivity; *RD*_*extra*_ = radial extra‐axonal diffusivity; RK = radial kurtosis; ROI = region of interest; SCQ = social communication questionnaire; SMA = supplementary motor areas; SQ = systemizing quotient; SRS‐2 = Social Responsiveness Scale‐2; *T*_*1*_ = longitudinal relaxation time; *T*_*2*_ = transverse relaxation time; VABS = Vineland Adaptive Behavior Scales; WAIS‐III = Wechsler Adult Intelligence Scale—Third Edition; WM = white matter.

### 4.1 Diffusion MRI Signal Representations

#### 4.1.1 Diffusion Kurtosis Imaging (DKI)

A total of 383 participants were included across six DKI studies (*N*_*autism*_ = 203, *M*_*age*_ = 2.7–38.9 years, 68% male; *N*_*neurotypical*_ = 180, *M*_*age*_ = 2.8–39.8 years, 65% male).

##### 4.1.1.1 Acquisition protocols and processing methods

All studies used 3 Tesla scanners, which provide a superior signal‐to‐noise ratio compared to 1.5 Tesla systems, from General Electric (GE; He et al. 2023; Shen et al. 2024; Tang et al. 2022), Philips (Hattori et al. 2019; Nagai et al. 2024), or Siemens (McKenna et al. 2020). Matrix sizes varied between 128 × 128 (Hattori et al. 2019; Nagai et al. 2024; Tang et al. 2022) and 256 × 256 (He et al. 2023; Shen et al. 2024), with in‐plane resolutions ranging from 0.78 mm × 0.78 mm (He et al. 2023; Shen et al. 2024) to 2.3 mm × 2.3 mm (McKenna et al. 2020), slice thicknesses from 2 mm (Hattori et al. 2019; Nagai et al. 2024) to 4 mm (He et al. 2023; Shen et al. 2024), and FOV from 200 mm × 200 mm (He et al. 2023; Shen et al. 2024) to 256 mm × 256 mm (Hattori et al. 2019; Nagai et al. 2024). Additionally, TRs ranged from 4500 ms (Tang et al. 2022) to 9810 ms (Hattori et al. 2019; Nagai et al. 2024) and TEs from 2.3 ms (He et al. 2023) to 100 ms (Hattori et al. 2019; Nagai et al. 2024). Despite these variations, *b*‐values of 1000 and 2000 s/mm^2^ were standard across all DKI studies, with the number of diffusion directions for each specific *b*‐value varying from 12 to 42 (McKenna et al. 2020). One study noted using a total of 30 directions, but the distribution for each *b*‐value remained unclear (Tang et al. 2022). Overall, imaging acquisition times ranged from approximately 5 min (Tang et al. 2022) to 13 min (Hattori et al. 2019; Nagai et al. 2024).

DKI studies commonly used the FMRIB Software Library (FSL; Jenkinson et al. 2012) tools for key processing tasks, such as motion and eddy current correction. Two studies also reported using additional tools, including Interactive Data Language (Exelis Visual Information Solutions, Boulder, Colorado; McKenna et al. 2020), in‐house developed code in MATLAB (McKenna et al. 2020), or the iQuant workstation (GE Healthcare, Beijing, China; He et al. 2023). DKI model fitting and metric estimation were commonly performed using a diffusional kurtosis estimator (Tabesh et al. 2011) implemented in MATLAB (Hattori et al. 2019; McKenna et al. 2020; Nagai et al. 2024). All studies derived AK, MK, and RK, with half also deriving FAK (He et al. 2023; Shen et al. 2024; Tang et al. 2022).

##### 4.1.1.2 Group differences and associations

Mixed group differences were observed, especially for AK, MK, and FAK. For example, several studies found lower AK in WM multi‐fibers (Hattori et al. 2019; He et al. 2023) and in GM regions (e.g., frontal lobe, caudate; Tang et al. 2022) in autism, suggesting that autistic individuals may have altered axonal organization or less directional coherence compared to neurotypical controls. Conversely, other studies reported higher AK in WM tracts (e.g., forceps minor, fornix; Nagai et al. 2024; Shen et al. 2024). Similarly, while two studies showed lower MK in GM regions (e.g., frontal and temporal lobes; McKenna et al. 2020; Tang et al. 2022), suggesting reduced tissue complexity or fewer diffusion barriers in autistic individuals, two other studies found higher MK in WM tracts (Nagai et al. 2024; Shen et al. 2024). Additionally, although lower FAK in multi‐fiber WM regions has been reported in autism (He et al. 2023), suggesting reduced microstructural anisotropy and potentially less organized or less directionally complex tissue, higher FAK was also observed in both multi‐fiber tracts (e.g., fornix, corpus callosum; Shen et al. 2024) and GM regions (e.g., frontal lobe, hippocampus; Tang et al. 2022). Regarding RK, lower RK was found in GM regions in autism (McKenna et al. 2020; Tang et al. 2022), suggesting atypical myelination or radial tissue organization compared to neurotypical controls.

Results regarding associations with developmental outcomes in the autism group were also mixed. Three studies found that lower AK, MK, and RK are associated with more negative developmental outcomes (Hattori et al. 2019; McKenna et al. 2020; Nagai et al. 2024). For instance, Hattori et al. found negative associations between AK in WM regions (e.g., inferior fronto‐occipital fasciculus) and the Japanese version of the Autism Spectrum Quotient (AQ; Wakabayashi et al. 2004), suggesting that altered axonal properties or reduced directional coherence are associated with greater autism symptom severity. Similarly, McKenna et al. reported that lower MK in GM regions (i.e., left entorhinal cortex, right parietal lobe) is associated with greater severity scores on the Restrictive Behaviors and Social Interactions subscales of the Autism Diagnostic Interview‐Revised (ADI‐R; Lord et al. 1994). Additionally, Nagai et al. found that lower RK in a WM cluster involving the splenium and body of the corpus callosum is associated with greater severity on the AQ‐Imagination subscale. Conversely, Hattori et al. found that lower AK in WM regions (e.g., inferior fronto‐occipital fasciculus) is associated with better outcomes on the Systemizing Quotient (SQ; Baron‐Cohen et al. 2003), and Nagai et al. also found that lower MK in WM tracts (e.g., right corticospinal tract) is associated with better outcomes on the Empathizing Quotient (EQ; Baron‐Cohen and Wheelwright 2004). Interestingly, although non‐significant group differences were reported, no non‐significant associations were observed in the DKI studies.

#### 4.1.2 Constrained Spherical Deconvolution (CSD)

A total of 864 participants were included across nine CSD studies (*N*_*autism*_ = 449, *M*_*age*_ = 4.2–39.2 years, 73% male; *N*_*control*_ = 415, *M*_*age*_ = 4.5–40 years, 70% male). While most controls were neurotypical (*N* = 361), 54 were autistic individuals from two studies that examined the impact of theta burst stimulation on WM macro‐ and microstructure in autistic individuals only, who were randomized into treatment groups.

##### 4.1.2.1 Acquisition protocols and processing methods

Imaging data were acquired using a 3 Tesla scanner from GE (Dimond et al. 2019; Guerrero‐Gonzalez et al. 2022; Kirkovski et al. 2023; Yeh et al. 2024), Siemens (Kirkovski et al. 2020, 2023; Ni et al. 2023; Yeh et al. 2022), or Philips (Dong et al. 2023; Kirkovski et al. 2023). CSD studies showed considerable variability in acquisition parameters, reflecting diverse goals and methodologies. Specifically, six reported matrix sizes ranged from 64 × 64 (Kirkovski et al. 2023) to 128 × 128 (Kirkovski et al. 2020, 2023); eight reported in‐plane resolutions between 1.875 mm × 1.875 mm (Kirkovski et al. 2023) and 3 mm × 3 mm (Kirkovski et al. 2023); FOV varied from 128 mm × 128 mm (Kirkovski et al. 2023) to 270 mm × 270 mm (Dong et al. 2023); and five studies reported slice thicknesses from 2 mm (Kirkovski et al. 2020, 2023; Newman et al. 2024) to 3.6 mm (Guerrero‐Gonzalez et al. 2022). TR and TE also varied widely, with 11 reported TRs ranging from 2238 ms (Yeh et al. 2022) to 20,244 ms (Kirkovski et al. 2023), and 10 reported TEs from 74 ms (Newman et al. 2024) to 102 ms (Kirkovski et al. 2020). For diffusion weighting, studies employing a single *b*‐value typically used either 2000 s/mm^2^ (Dimond et al. 2019; Kirkovski et al. 2020; Yeh et al. 2024) or 1000 s/mm^2^ (Kirkovski et al. 2023; Newman et al. 2024), while others utilized two *b*‐values (Ni et al. 2023) or three *b*‐values (Guerrero‐Gonzalez et al. 2022; Yeh et al. 2022). The number of directions ranged from nine (Guerrero‐Gonzalez et al. 2022) to 128 (Yeh et al. 2024). Notably, one study collected data across three sites, each with different parameters (Kirkovski et al. 2023). Overall, imaging acquisition time varied from approximately 4.5 min (Dong et al. 2023) to 24 min (Kirkovski et al. 2023), although many studies omitted this information.

Data processing was mainly carried out using FSL and MRtrix3 (Tournier et al. 2019). One study used an in‐house pipeline that combined both tools (TiDi‐Fused; Guerrero‐Gonzalez et al. 2022), while two studies followed a recommended fixel‐based analysis pipeline (Dhollander et al. 2021; Raffelt et al. 2017) using both tools (Kirkovski et al. 2020, 2023). Additionally, some studies performed single‐shell 3‐tissue (Dimond et al. 2019; Dong et al. 2023; Kirkovski et al. 2020, 2023; Newman et al. 2024) or multi‐shell multi‐tissue CSD (Guerrero‐Gonzalez et al. 2022; Ni et al. 2023; Yeh et al. 2022, 2024) using MRtrix3 to estimate fiber orientation distribution and derive fixel metrics: FD (Dimond et al. 2019; Dong et al. 2023; Guerrero‐Gonzalez et al. 2022; Kirkovski et al. 2020, 2023; Ni et al. 2023; Yeh et al. 2022, 2024); FC (Dimond et al. 2019; Dong et al. 2023; Kirkovski et al. 2020, 2023; Ni et al. 2023; Yeh et al. 2022, 2024); and FDC (Dimond et al. 2019; Dong et al. 2023; Kirkovski et al. 2020, 2023; Newman et al. 2024; Ni et al. 2023; Yeh et al. 2022, 2024). Intriguingly, one study used FD as a key component in calculating the *g*‐ratio and conduction velocity (Newman et al. 2024).

##### 4.1.2.2 Group differences and associations

Consistent reductions in FD (Dimond et al. 2019; Kirkovski et al. 2023; Yeh et al. 2022), FC (Kirkovski et al. 2023; Yeh et al. 2022), and FDC (Kirkovski et al. 2020, 2023; Yeh et al. 2022) have been observed in autism. This suggests potentially lower levels of axonal development, tract maturation, or broader alterations in micro‐ and macrostructural organization in autistic individuals compared to neurotypical controls. For example, Dimond et al. reported lower FD in the WM tracts (e.g., the corpus callosum, right arcuate fasciculus). Similarly, Yeh et al. found lower FDC in the right cerebellum Crus II, and Kirkovski et al. (2020) observed lower FDC in autistic females compared to neurotypical females.

Regarding associations with developmental outcomes in the autism group, only negative associations were observed for all three fixel metrics, indicating that lower axonal density, smaller tract size, and alterations in micro‐ and macrostructural organization are linked to greater developmental challenges in autistic individuals. Specifically, FD in the splenium of the corpus callosum was negatively associated with the Social Responsiveness Scale‐2 (SRS‐2; Constantino 2012; Dimond et al. 2019), and FD in the dorsolateral prefrontal cortex was negatively associated with the Behavior Rating Inventory of Executive Function‐Global Executive Composite (BRIEF‐GEC; Gioia et al. 2000; Yeh et al. 2022). Additionally, Yeh et al. found a negative association between FC in the right cerebellum Crus I and the Autism Diagnostic Observation Schedule‐2 calibrated severity scores (ADOS‐2 CSS; Hus and Lord 2014), as well as a negative association between FDC in the dorsolateral prefrontal cortex and BRIEF‐GEC. Although there was notable consistency, non‐significant group differences and associations were also reported.

### 4.2 Diffusion MRI Biophysical Models

#### 4.2.1 Neurite Orientation Dispersion and Density Imaging (NODDI)

A total of 826 participants were included across nine NODDI studies (*N*_*autism*_ = 404, *M*_*age*_ = 8.7–32.9 years, 86% male; *N*_*neurotypical*_ = 424, *M*_*age*_ = 8.2–34.4 years, 83% male).

##### 4.2.1.1 Acquisition protocols and processing methods

All studies acquired imaging data using 3 Tesla scanners from Siemens (DiPiero, Cordash, et al. 2023; Kitamura et al. 2023a, 2023b; Matsuoka et al. 2020; Yasuno et al. 2020), GE (DiPiero, Surgent, et al. 2023; Travers et al. 2024), or Philips (Andica et al. 2021; Arai et al. 2023). Imaging parameters varied across NODDI studies. For instance, four different matrix sizes were reported, with 114 × 114 being the most common (Kitamura et al. 2023a, 2023b; Matsuoka et al. 2020; Yasuno et al. 2020), along with in‐plane resolutions and slice thicknesses typically measuring 2 mm × 2 mm and 2 mm, respectively (Andica et al. 2021; Arai et al. 2023; DiPiero, Cordash, et al. 2023; Kitamura et al. 2023a, 2023b; Matsuoka et al. 2020; Yasuno et al. 2020). Additionally, FOV ranged from 224 × 224 mm (Yasuno et al. 2020) to 256 × 256 mm (Andica et al. 2021; Arai et al. 2023; DiPiero, Surgent, et al. 2023); TRs varied from 4870 ms (DiPiero, Cordash, et al. 2023) to 17,500 ms (Kitamura et al. 2023a, 2023b; Matsuoka et al. 2020; Yasuno et al. 2020); and TEs ranged from 74.4 ms (DiPiero, Surgent, et al. 2023; Travers et al. 2024) to 100 ms (Andica et al. 2021; Arai et al. 2023). Notably, one study applied different parameters for each cohort (DiPiero, Surgent, et al. 2023). Most studies used two *b*‐values of 1000 and 2000 s/mm^2^ (Andica et al. 2021; Arai et al. 2023; Kitamura et al. 2023a, 2023b; Matsuoka et al. 2020; Yasuno et al. 2020), while a few studies used three *b*‐values (*b* = 350, 800, 2000 s/mm^2^; DiPiero, Surgent, et al. 2023; Travers et al. 2024) or four *b*‐values (*b* = 350, 1000, 2000, 3000 s/mm^2^; DiPiero, Cordash, et al. 2023). For specific *b*‐values, diffusion directions ranged from as few as nine directions (DiPiero, Surgent, et al. 2023; Travers et al. 2024) to 96 directions (DiPiero, Cordash, et al. 2023), with 30 directions being the most common (Kitamura et al. 2023a, 2023b; Matsuoka et al. 2020; Yasuno et al. 2020). Overall, imaging acquisition time varied from approximately 10 min (DiPiero, Surgent, et al. 2023) to 20 min (Matsuoka et al. 2020). It is important to note that some studies did not report all parameter details.

Diffusion‐weighted imaging processing primarily used tools from FSL, including EDDY (Andersson et al. 2016, 2017) and FLIRT (Jenkinson et al. 2002). Additionally, three studies also used MRtrix3 (DiPiero, Surgent, et al. 2023; DiPiero, Cordash, et al. 2023; Travers et al. 2024) along with Advanced Normalization Tools (DiPiero, Cordash, et al. 2023; Tustison et al. 2021). These studies specifically employed an in‐house pipeline (i.e., TiDi‐Fused), which integrates FSL and MRtrix3. NODDI model fitting and metric estimation for NDI, ODI, and FISO (Andica et al. 2021; Arai et al. 2023; DiPiero, Surgent, et al. 2023; DiPiero, Cordash, et al. 2023) were often performed using the NODDI toolbox in MATLAB (Andica et al. 2021; Kitamura et al. 2023a, 2023b; Matsuoka et al. 2020; Yasuno et al. 2020).

##### 4.2.1.2 Group differences and associations

Although mixed results were observed regarding group differences across three NODDI metrics, there was greater consensus for NDI. Most studies reported lower NDI in autism (Andica et al. 2021; Arai et al. 2023; DiPiero, Surgent, et al. 2023; DiPiero, Cordash, et al. 2023; Yasuno et al. 2020), suggesting lower neurite density or differences in neurite organization in autistic individuals compared to neurotypical controls. For example, Andica et al. found lower NDI in WM tracts, while DiPiero, Surgent et al. found lower NDI in GM regions. Additionally, Yasuno et al. reported smaller NDI (in clusters including the ventral occipital complex region encompassing the fusiform gyrus, inferior parietal region, and forceps major of the corpus callosum) in autistic individuals with a higher index of difference in correctness between vertical and horizontal slits (IDCS), which measures facial emotional recognition, compared to neurotypical individuals. Only one study found higher NDI in the right superior temporal gyrus (Kitamura et al. 2023a). Similar to Yasuno et al., Kitamura et al. observed group differences after stratifying the autism group based on the severity of sensory over‐responsivity, measured by the Japanese version of the Adolescent/Adult Sensory Profile‐Sensory Over‐Responsivity (AASP‐SOR; Brown and Dunn 2002; Ito et al. 2013); in this case, higher NDI was found in autistic individuals with a higher AASP‐SOR.

Three studies reported significant differences in ODI, with higher ODI observed in autism across widespread WM tracts (DiPiero, Cordash, et al. 2023) and in the GM region of the left occipital gyrus (Matsuoka et al. 2020), indicating greater neurite dispersion, reflecting more variable neurite orientations, compared to neurotypical controls. Intriguingly, autistic individuals with higher IDCS showed smaller ODI in clusters that include the ventral occipital complex region encompassing the fusiform gyrus, inferior parietal region, and forceps major of the corpus callosum (Yasuno et al. 2020). Regarding FISO, higher FISO in the WM tracts was reported in autism (Andica et al. 2021), suggesting greater extracellular free water content compared to neurotypical individuals.

Mixed results were observed regarding associations with NDI in the autism group. Two studies reported that higher NDI is associated with more negative developmental outcomes (Kitamura et al. 2023a, 2023b), while two other studies found that higher NDI is associated with better developmental outcomes (Arai et al. 2023; Yasuno et al. 2020). For instance, Kitamura et al. (2023a) found positive associations between NDI in the right superior temporal gyrus and both AASP‐SOR and the Japanese version of the Child Abuse Trauma Scale (CATS; Sanders and Becker‐Lausen 1995; Tanabe et al. 2010), suggesting that higher neurite density is associated with more severe sensory symptoms and trauma. Conversely, Arai et al. reported an association between NDI in GM regions (e.g., left rostral middle frontal, superior frontal) and EQ, indicating that higher neurite density is associated with better empathizing ability. Similarly, Yasuno et al. observed an association between NDI (in clusters including the ventral occipital complex region, superior temporal/parietal association areas, and forceps major of the corpus callosum) and IDCS scores, suggesting that higher neurite density relates to better facial emotion recognition.

Association results for ODI were also mixed, suggesting that higher ODI is associated with both negative (Matsuoka et al. 2020; Travers et al. 2024) and positive developmental outcomes (DiPiero, Surgent, et al. 2023; Yasuno et al. 2020). For example, Matsuoka et al. found an association between ODI in the left occipital gyrus and both AASP‐Low Registration and AASP‐Passive Behavioral Response, suggesting that a greater dispersion of neurite orientations is associated with greater sensory over‐responsivity. Similarly, Travers et al. observed an association between ODI in the bilateral parvicellular reticular formation alpha cluster and a summary measure of the Repetitive Behavior Scale‐Revised (RBS‐R; Bodfish et al. 1999), Social Communication Questionnaire (SCQ; Rutter et al. 2003), and SRS‐2, indicating that a greater dispersion of neurite orientations is associated with greater autism symptom severity. Notably, the association found by Travers et al. reflects a summary measure of ODI and DTI metrics. In contrast, DiPiero, Surgent et al. found an association between ODI in GM areas, predominantly located in the right hemisphere, and the ADOS‐2 CSS, suggesting that greater alignment of neurite orientation is associated with greater autism symptom severity. No associations were observed for FISO, and importantly, no group differences or associations were reported as well.

#### 4.2.2 White Matter Tract Integrity (WMTI)

A total of 172 participants were included across three WMTI studies (*N*_*autism*_ = 94, *M*_*age*_ = 3–21.4 years, 82% male; *N*_*neurotypical*_ = 78, *M*_*age*_ = 3.0–21.7 years, 78% male).

##### 4.2.2.1 Acquisition protocols and processing methods

All studies used 3 Tesla scanners from Siemens (Lazar et al. 2014; Sui et al. 2018) or GE (Shen et al. 2024). Only one study (Shen et al. 2024) reported matrix size, which was 256 × 256, with in‐plane resolutions of either 0.78 mm × 0.78 mm (Shen et al. 2024) or 2.3 mm × 2.3 mm (Lazar et al. 2014; Sui et al. 2018). Slice thicknesses were either 2.3 mm (Lazar et al. 2014; Sui et al. 2018) or 4 mm (Shen et al. 2024), and only one study (Shen et al. 2024) reported a 200 mm × 200 mm FOV. Despite these differences, TRs were either 8100 ms (Lazar et al. 2014; Sui et al. 2018) or 8200 ms (Shen et al. 2024), with an approximate 97 ms TE across all studies. Similarly, *b*‐values of 1000 and 2000 s/mm^2^ were standard in all WMTI studies, with the number of diffusion directions varying from 12 to 42 for each *b*‐value (Lazar et al. 2014; Sui et al. 2018). Overall, only one study reported an imaging acquisition time of 7 min 23 s (Shen et al. 2024).

WMTI studies consistently used FSL tools for key processing tasks, with one study also reporting the use of additional tools, including Interactive Data Language (Exelis Visual Information Solutions, Boulder, Colorado; Sui et al. 2018) and in‐house developed code in MATLAB (Sui et al. 2018). Diffusion parameters were calculated using either a diffusional kurtosis estimator (Tabesh et al. 2011) implemented in MATLAB (Lazar et al. 2014; Sui et al. 2018) or the PyDesigner method (Dhiman et al. 2021; Shen et al. 2024) to derive *f*_*axon*_, *D*_*axon*_, *AD*_*extra*_, *RD*_*extra*_ (Lazar et al. 2014; Shen et al. 2024; Sui et al. 2018), and tortuosity (Shen et al. 2024).

##### 4.2.2.2 Group differences and associations

Different group differences were observed for most WMTI metrics, except *RD*_*extra*_, where all three studies found no differences between the autism and neurotypical groups. Two studies reported lower *f*_*axon*_ in most major WM tracts, including the corpus callosum, corticospinal tracts, and superior longitudinal, inferior longitudinal, and inferior fronto‐occipital fasciculi (Lazar et al. 2014), mainly in the midbody, isthmus, and splenium of the corpus callosum (Sui et al. 2018), and lower *D*_*axon*_ in some WM regions (Lazar et al. 2014), mostly in the midbody, isthmus, and splenium of the corpus callosum (Sui et al. 2018). However, one study reported higher *f*_*axon*_ and *D*_*axon*_ primarily in the corpus callosum and fornix in the autism group (Shen et al. 2024). Regarding *AD*_*extra*_, one study found lower *AD*_*extra*_ in a localized area of the prefrontal corpus callosum (Lazar et al. 2014), another found higher *AD*_*extra*_ primarily in the corpus callosum and fornix (Shen et al. 2024), and a third reported no group differences in *AD*_*extra*_ (Sui et al. 2018). Notably, one study observed higher tortuosity in the autism group, suggesting denser axonal packing or altered fiber organization compared to neurotypical controls.

Similar to group differences, results regarding associations with developmental outcomes in the autism group were mixed. One study observed a positive association between Wechsler Adult Intelligence Scale‐III (WAIS‐III) Digit Symbol‐Coding and *f*_*axon*_ and *D*_*axon*_ in the left inferior longitudinal and left fronto‐occipital fasciculi (Lazar et al. 2014), suggesting that greater axonal density or integrity is associated with better processing speed in autistic individuals. Conversely, another study found no association between WMTI metrics and WAIS‐III Digit Symbol‐Coding or Symbol Search in the mid and posterior segments (Sui et al. 2018).

### 4.3 Relaxometry

A total of 390 participants were included across seven relaxometry studies (*N*_*autism*_ = 221, *M*_*age*_ = 3–26.5 years, 84% male; *N*_*control*_ = 169, *M*_*age*_ = 3.0–27.9 years, 75% male). Notably, the control group consisted of individuals who were neurotypical (*N* = 136), globally developmentally delayed (*N* = 17), or developmentally delayed (*N* = 16).

#### 4.3.1 Acquisition Protocols and Processing Methods

Most studies used a 3 Tesla scanner (Hasan et al. 2013; Hendry et al. 2006; Li et al. 2023; Surgent et al. 2024), while some employed a 1.5 Tesla scanner (Deoni et al. 2015; Petropoulos et al. 2006) or a 7 Tesla scanner (Fischi‐Gomez et al. 2021). Various sequences and techniques were used. Protocols measuring *T*_*1*_ used DESPOT1/mcDESPOT (Deoni et al. 2015), MP2RAGE (Fischi‐Gomez et al. 2021), SyMRI/MAGiC (Li et al. 2023), or MPnRAGE (Surgent et al. 2024). *T*_*2*_ estimation relied on DESPOT2/mcDESPOT (Deoni et al. 2015), dual fast spin echo (Hasan et al. 2013), Gradient Echo Sampling of the Free Induction Decay and Echo (Hendry et al. 2006), SyMRI/MAGiC (Li et al. 2023), or fast spin echo sequences (Petropoulos et al. 2006). *T*_*2*_ *'* was calculated using Gradient Echo Sampling of the Free Induction Decay and Echo (Hendry et al. 2006), while MWF (Deoni et al. 2015) was derived from mcDESPOT (Deoni et al. 2015).

Data processing further contributed to heterogeneity among relaxometry studies. Some studies used well‐known software tools, such as FSL and SPM2, while others employed alternative platforms like Advanced Normalization Tools, SyMRI, or custom/in‐house pipelines (i.e., homomorphic filtering; Guillemaud 1998) and software (Deoni et al. 2013). *T*_*2*_ was the most commonly quantified parameter (Deoni et al. 2015; Hasan et al. 2013; Hendry et al. 2006; Li et al. 2023; Petropoulos et al. 2006), followed by *T*_*1*_ (Deoni et al. 2015; Fischi‐Gomez et al. 2021; Li et al. 2023; Surgent et al. 2024), *T*_*2*_ *'* (Hendry et al. 2006), and MWF (Deoni et al. 2015).

#### 4.3.2 Group Differences and Associations

Inconsistent differences in relaxation times were observed across studies. For example, one study reported shorter *T*_*1*_ in both WM tracts (the genu and splenium of the corpus callosum) and GM structure (the right thalamus) in the autism group compared to the global developmental delay group (Li et al. 2023), suggesting greater myelin content or more organized tissue in autistic individuals. Conversely, another study found longer *T*_*1*_ bilaterally in WM tracts (e.g., the internal capsule) and GM structure (the thalamus) in the autism group compared to neurotypical controls (Deoni et al. 2015). Similarly, two studies reported shorter *T*_*2*_ in both WM and GM in the autism group compared to the global developmental delay group (Li et al. 2023) and the developmental delay group (Petropoulos et al. 2006), suggesting more myelinated or denser tissue in autistic individuals. Meanwhile, some other studies reported longer *T*_*2*_ in WM (Hendry et al. 2006) and GM (Petropoulos et al. 2006) in the autism group compared to neurotypical controls. Additionally, lower MWF was observed in WM (e.g., the internal capsule, the cingulum) and GM (e.g., the caudate nuclei) in the autism group (Deoni et al. 2015), suggesting lower myelin content or delayed myelination in autistic individuals compared to neurotypical individuals.

Several mixed associations with developmental outcome measures were reported in the autism group. Specifically, a positive association was found between the inverse of *T*_*1*_ (i.e., *R*_*1*_) and grip strength, measured by the Jamar hand dynamometer (Heaton et al. 1991; Surgent et al. 2024), suggesting that greater tissue myelination is associated with stronger grip strength in autistic individuals. Similarly, the reported negative association between MWF and ADOS total score, ADOS‐Social Interaction, and AQ (Deoni et al. 2015) suggests that lower myelin content is associated with greater autism symptom severity. However, *T*_*2*_ in both WM (periventricular WM, genu of the corpus callosum) and GM (left thalamus) was positively associated with the Gesell Developmental Scale of Gross Motor (i.e., DQ2) and Personal‐Social Behavior (i.e., DQ5; Li et al. 2023), indicating that higher water content or less mature tissue characteristics are associated with better motor and social outcomes in autistic individuals. Notably, considerable non‐significant group differences and some non‐significant associations were also reported.

### 4.4 Magnetization Transfer Imaging (MTI)

A total of 136 participants were included in a study that used MTI to assess the myelination of the corpus callosum (*N*_*autism*_ = 101, *M*_*age*_ = 4.5 years, 81% male; *N*_*neurotypical*_ = 35, *M*_*age*_ = 4.0 years, 66% male; Gozzi et al. 2012).

#### 4.4.1 Acquisition Protocols and Processing Methods

Gozzi et al. used a 1.5 Tesla GE scanner to acquire MTI data with a matrix size of 256 × 192, an in‐plane resolution of 0.94 mm × 1.25 mm, a slice thickness of 3.0 mm, a FOV of 240 mm × 240 mm, and TR and TE of 500 ms and 12 ms, respectively. The imaging acquisition time was not reported. Data processing was conducted using the Medical Image Processing, Analysis, and Visualization software (McAuliffe et al. 2001) and in‐house software (Ostuni et al. 2002) to estimate MTI metrics.

#### 4.4.2 Group Differences and Associations

Gozzi et al. revealed that all three MTI metrics in the corpus callosum—mean MTR, peak location, and peak height of the MTR histogram—were higher in autistic children than in their neurotypical peers, indicating greater myelin content and tissue homogeneity in the corpus callosum in autism. The association with nonverbal DQ was also examined, but no correlation was found.

### 4.5 Similarities and Differences in Brain Regions Across Techniques

A total of 13 brain regions were identified, with group differences examined using at least two techniques. Notably, considerable variability in the analyzed regions across techniques limited the ability to synthesize findings regarding associations.

The corpus callosum was the only region examined using all techniques. Specifically, CSD (FD, FC, FDC; Dimond et al. 2019; Kirkovski et al. 2023; Yeh et al. 2022), DKI (AK; Hattori et al. 2019), NODDI (NDI; Andica et al. 2021; DiPiero, Cordash, et al. 2023), WMTI (*f*_*axon*_, *AD*_*extra*_, *D*_*axon*_; Lazar et al. 2014; Sui et al. 2018), and relaxometry (*T*_*1*_, *T*_*2*_; Li et al. 2023) revealed lower values in the corpus callosum for autistic individuals. Conversely, DKI (AK, MK, FAK; Shen et al. 2024), WMTI (*f*_*axon*_, *AD*_*extra*_, *D*_*axon*_, tortuosity; Shen et al. 2024), MTI (mean MTR, peak location/height of the MTR histogram; Gozzi et al. 2012), and NODDI (ODI, FISO; Andica et al. 2021; DiPiero, Cordash, et al. 2023) showed higher values in the corpus callosum. Notably, AK of DKI and *f*_*axon*_, *AD*_*extra*_, and *D*_*axon*_ of WMTI exhibited conflicting group differences.

Group differences across four regions were examined using three techniques. In autistic individuals, lower values were observed in the internal capsule using NODDI (NDI; Andica et al. 2021; DiPiero, Cordash, et al. 2023) and relaxometry (MWF; Deoni et al. 2015); conversely, other metrics of NODDI (ODI; DiPiero, Cordash, et al. 2023) and relaxometry (*T*_*1*_; Deoni et al. 2015), along with DKI (AK, MK, FAK; Nagai et al. 2024; Shen et al. 2024), showed higher values in the internal capsule. In the fornix, autistic individuals displayed lower NODDI (NDI; DiPiero, Cordash, et al. 2023) values, while other metrics of NODDI (ODI; DiPiero, Cordash, et al. 2023), DKI (AK, FAK; Shen et al. 2024), and WMTI (*f*_*axon*_, *AD*_*extra*_, *D*_*axon*_, tortuosity; Shen et al. 2024) indicated higher values. In the superior longitudinal fasciculus, NODDI (NDI; Andica et al. 2021; DiPiero, Cordash, et al. 2023) and WMTI (*f*_*axon*_; Lazar et al. 2014) showed lower values, whereas other NODDI metrics (ODI, FISO; Andica et al. 2021; DiPiero, Cordash, et al. 2023) were higher; AK from DKI produced mixed results, with one study observing lower AK (He et al. 2023) and another reporting higher AK (Nagai et al. 2024). In the inferior fronto‐occipital fasciculus, NODDI (NDI; Andica et al. 2021), CSD (FD; Dimond et al. 2019), and WMTI (*f*_*axon*_; Lazar et al. 2014) exhibited lower values, while another NODDI metric (FISO; Andica et al. 2021) showed higher values in autistic individuals.

Group differences in the remaining eight regions were examined using two techniques, with five regions using DKI and NODDI. In autistic individuals, in the anterior corona radiata, NDI was lower (DiPiero, Cordash, et al. 2023), while ODI (DiPiero, Cordash, et al. 2023), AK, and MK were higher (Nagai et al. 2024). In the anterior thalamic radiation, NDI (Andica et al. 2021) and AK were lower (He et al. 2023), whereas MK was higher (Nagai et al. 2024). In the cerebral peduncle, ODI (DiPiero, Cordash, et al. 2023) and AK were higher (Shen et al. 2024). In both the frontal and temporal lobes, NDI (Arai et al. 2023; DiPiero, Surgent, et al. 2023) and AK, MK, and RK (McKenna et al. 2020; Tang et al. 2022) were lower, while FAK was higher.

Using NODDI and CSD, lower NDI (Andica et al. 2021; DiPiero, Cordash, et al. 2023) and FD (Dimond et al. 2019) were observed in the uncinate fasciculus. Using DKI and relaxometry, lower AK and MK (Tang et al. 2022) and MWF (Deoni et al. 2015) were observed in the caudate nucleus. Lastly, in the cerebellum, CSD and relaxometry revealed lower FD and FC (Yeh et al. 2022) and MWF (Deoni et al. 2015), along with higher *T*_*1*_ (Deoni et al. 2015), in autistic individuals.

## 5 Discussion

This review provides an overview of the current landscape of advanced quantitative microstructure imaging techniques in autism research. Among the techniques reviewed, CSD and NODDI were identified as the most frequently utilized, whereas MTI was the least used. Despite our intention to include CHARMED, no studies utilizing this technique in autism research were identified. The preference for CSD and NODDI may be attributed to their feasible implementation within reasonable acquisition durations and their ability to provide detailed insights into neurite orientation, density, and WM pathways. Conversely, CHARMED relies on substantially higher *b*‐values (often up to 10,000 s/mm^2^) and more extensive multi‐shell acquisitions (Assaf and Basser 2005), rendering it less practical for many clinical and research settings, particularly for the autism population. Coupled with the limited evidence of added value for autism research, these demanding requirements may explain the underutilization of CHARMED and, similarly, MTI in autism research.

### 5.1 Variability Observed in Methodological Features

Most studies employed a 3 Tesla MRI scanner, which is considered standard in neuroimaging research due to its optimal balance of resolution and signal‐to‐noise ratio. Only one study employed a 7 Tesla scanner for *T*_*1*_ relaxometry (Fischi‐Gomez et al. 2021), while a few studies utilized 1.5 Tesla scanners for relaxometry (Deoni et al. 2015; Petropoulos et al. 2006) and MTI (Gozzi et al. 2012). This consistency in magnetic field strength reduces the likelihood that such differences account for variability in findings, allowing for a greater focus on acquisition parameters and processing methods. Notably, acquisition parameters varied widely, particularly in TR, TE, and the number of diffusion directions, which are key factors influencing the quality of diffusion modeling and the signal‐to‐noise ratio. For instance, shorter TR values can lower the signal‐to‐noise ratio, while longer TR values improve signal recovery but increase motion artifacts. Nevertheless, diffusion MRI studies revealed greater consistency in processing methods, frequently using FSL and MRtrix for preprocessing steps like eddy current and distortion correction. This uniformity mitigates some of the acquisition‐related variability, enhancing the reliability of the derived metrics. In relaxometry studies, however, heterogeneous relaxometry techniques and processing pipelines may partly explain variability in reported relaxation times. Harmonizing acquisition protocols and processing workflows remains essential for enhancing reproducibility and interpretability in autism research.

### 5.2 Microstructural Differences Observed Across Techniques

Findings across diffusion techniques, including CSD and NODDI, revealed relatively consistent patterns of altered neurodevelopmental processes in autistic individuals, despite methodological differences among studies. CSD studies frequently indicated reductions in FD, FC, and FDC, suggesting differences in the coherence and organization of WM fibers that support social communication and executive functioning. These reductions could reflect alterations in axonal organization or fiber bundle connectivity, underscoring vulnerabilities in long‐range neural pathways essential for cognitive integration. Notably, many of these findings were derived from studies focused on early to late adolescents, highlighting adolescence as a sensitive period of heightened brain plasticity (Fuhrmann et al. 2015), when delays or deviations in overall neurodevelopmental trajectories, including brain network maturation, may be particularly pronounced in autism (Cai et al. 2021). Similarly, NODDI studies commonly reported lower NDI, especially in autistic adults, suggesting lower neurite density and differences in the structural architecture supporting synaptic communication and large‐scale network formation. Conversely, findings regarding ODI were more variable, possibly reflecting the heterogeneity inherent in autism. Higher ODI may indicate either increased structural complexity or greater variability in neurite orientation and has been linked to specific challenges such as facial emotion recognition (Yasuno et al. 2020). This suggests that the extent and pattern of dispersion may have functional relevance depending on brain region and behavioral domain. DKI and WMTI findings were more heterogeneous overall, likely attributable to the broader age ranges of participants and differences in analytical focus. In particular, inconsistencies in FAK may reflect sensitivity to early neurodevelopmental processes such as myelination and axonal branching, which are especially dynamic in early childhood. Therefore, FAK and related metrics (AK, MK, RK) may represent distinct aspects of microstructural maturation at different developmental stages. Similarly, age or developmental stage may account for discrepancies observed in WMTI metrics, with lower values observed in autistic adults and higher values in autistic children, especially given that these patterns were found in largely overlapping regions such as the corpus callosum.

Findings from relaxometry and MTI further emphasize myelination as a sensitive and developmentally nuanced marker of brain structure in autism. While shorter *T*_*1*_ and *T*_*2*_ values are generally considered indicative of increased myelin content, conflicting results across studies likely reflect differences in imaging protocols, analytic approaches, and control group compositions. For example, some comparisons involved children with global developmental delay rather than neurotypical controls, potentially complicating interpretation. Moreover, elevated values of MTI metrics in the corpus callosum of autistic children, such as higher mean MTR and peak location, may suggest greater myelin content or macromolecular density; however, the limited number of MTI studies precludes firm conclusions.

### 5.3 Microstructural Relationships With Developmental Outcomes Observed Across Techniques

Relatively consistent relationships observed with developmental outcomes using advanced diffusion MRI highlight the role of specific microstructural features in shaping cognitive, emotional, and social behaviors, particularly during late adolescence and young adulthood. For instance, higher NDI in areas like the prefrontal cortex and superior temporal gyrus has been linked to better empathic ability in autistic adults. This indicates that a denser axonal and dendritic structure in regions critical for higher‐order cognition may promote more adaptive social functioning. Conversely, lower NDI in sensory processing areas, such as the ventral occipital complex, has been associated with greater sensory over‐responsiveness and poorer facial emotion recognition in young autistic adults, possibly reflecting disruptions in the development of sensory pathways that contribute to atypical perceptual experiences in autism. Fixel‐based analysis further illustrates the importance of WM organization during late adolescence. Reductions in FD and related metrics within WM tracts like the corpus callosum and dorsolateral prefrontal cortex seem to reflect lower fiber density and altered directional organization, which may hinder the neural communication necessary for social responsiveness and executive functioning. These alterations in both micro‐ and macrostructural integrity may underlie the challenges in higher‐order cognition observed in autism. Lower diffusion kurtosis values, as seen in AK, RK, and MK metrics, also correspond with increased social and emotional challenges, particularly in young autistic adults. Such reductions may indicate differences in microstructural environments, potentially due to variations in axonal and cellular architecture. These microstructural changes could impair the flexibility of WM networks necessary for effective emotional regulation and social interaction.

Relaxometry findings underscore the importance of developmental timing in tissue maturation and its impact on functional outcomes. In young autistic children, higher *R*_*1*_ values, typically associated with greater myelin content, corresponded with stronger grip strength, whereas longer *T*_*2*_ values were linked to better motor coordination and social engagement. Although longer *T*_*2*_ often indicates less mature tissue, these associations may suggest that a slower or more extended maturation trajectory in certain regions facilitates adaptive outcomes during early development. Conversely, in young autistic adults, lower MWF was associated with greater autism symptom severity, suggesting that less adequate myelination may become more maladaptive as the brain matures. These findings suggest that both the quantity and timing of myelination are critical factors, with distinct implications at different developmental stages. Interestingly, no such associations were observed for MTI metrics in young autistic children. While MTI is sensitive to myelin and macromolecular content, the absence of its association with behavioral outcomes in early development could reflect either a reduced sensitivity of this metric at this age or a lesser direct impact on behavior relative to relaxometry metrics.

### 5.4 Microstructural Differences Observed Across Brain Regions

The corpus callosum emerged as a key region of interest across all techniques, underscoring its central role in autism‐related WM alterations and reinforcing existing evidence of atypical interhemispheric connectivity in autistic individuals (e.g., reduced FA; Alexander, Lee, Lazar, Boudos, et al. 2007; Alexander, Lee, Lazar, and Field 2007; Travers et al. 2012, 2015). Relatively consistent reductions observed through diffusion MRI techniques—including lower fiber density and fiber cross‐section (CSD), kurtosis‐based complexity (DKI), compartmental‐model‐based specificity (WMTI), and neurite density (NODDI)—suggest differences in axonal coherence, microstructural complexity, and the density of neural projections in the corpus callosum. Relaxometry findings indicating prolonged *T*_*1*_ and *T*_*2*_ values may additionally point to delayed or atypical maturation of tissue properties, such as myelination. In contrast, elevated MTI metrics in the corpus callosum may reflect increased macromolecular content, which could indicate greater myelination or possibly atypical compensatory myelin changes. Collectively, these converging findings implicate the corpus callosum in disrupted interhemispheric communication in autism and highlight the value of multimodal approaches for understanding WM pathology.

Beyond the corpus callosum, further alterations were observed in various WM tracts and subcortical regions, such as the anterior corona radiata, anterior thalamic radiation, fornix, inferior fronto‐occipital fasciculus, uncinate fasciculus, and cerebellum. Findings across these regions suggest region‐specific disruptions in WM development. Widespread reductions in NDI suggest decreased neurite density, reflecting fewer or less mature axons and dendrites. In many of these same regions, elevated ODI points to increased dispersion of fiber orientations, which may reflect either greater structural complexity or more variable fiber architecture, contingent upon regional context and developmental timing.

These findings collectively support the notion that specific alterations in WM microstructure—such as decreased axonal density, increased variability in fiber orientation, or delayed tissue maturation—may interfere with the efficiency and specialization of neural networks. This disruption potentially contributes to the atypical neurodevelopmental trajectories observed in autism. Furthermore, the findings highlight the potential of integrating diffusion MRI, relaxometry, and MTI techniques to capture the nuanced and regionally variable nature of these microstructural changes. Importantly, this comprehensive perspective reinforces the view that differences in WM organization are central features of autism, with possible implications for cognitive, sensory, and socio‐emotional functioning across various developmental stages.

## 6 Limitations of Advanced Quantitative Microstructure Imaging Techniques

Diffusion MRI signal representations, such as DKI and CSD, enhance the limitations of DTI by increasing sensitivity to complex tissue structures, although each has notable limitations. DKI captures non‐Gaussian diffusion to characterize tissue complexity; however, its metrics are nonspecific, reflecting diffusion heterogeneity without clarifying its source (Jensen et al. 2005; Steven et al. 2014). Additionally, DKI requires high *b*‐values and extended acquisition times, which may constrain its clinical utility. CSD advances fiber orientation mapping in regions with crossing fibers, but standard single‐tissue implementations are susceptible to inaccuracies due to partial volume effects from GM or cerebrospinal fluid (Dhollander and Connelly 2016). Although multi‐tissue CSD mitigates this concern, it requires more advanced acquisition protocols. Furthermore, spherical deconvolution is inherently ill‐posed and sensitive to noise, thereby necessitating regularization to stabilize the estimates (Tournier et al. 2007).

Diffusion MRI biophysical models, such as CHARMED, NODDI, and WMTI, offer meaningful biological interpretations of microstructural characteristics; however, they rely on simplifying assumptions that could restrict their accuracy or not fully characterize the complexity of the underlying biology. CHARMED models axons as impermeable cylinders and assumes negligible water exchange between compartments (Assaf et al. 2004, 2008; Assaf and Basser 2005). While such simplification facilitates modeling, it does not fully capture the complexity inherent in brain tissue. Additionally, it requires high *b*‐values and multi‐shell acquisitions, which may increase scan time and reduce the signal‐to‐noise ratio. NODDI offers biologically interpretable measures of neurite density and orientation dispersion; nonetheless, it relies on fixed diffusivity assumptions and idealized geometries (Zhang et al. 2012), which may compromise accuracy in heterogeneous tissues. Although NODDI incorporates an isotropic compartment to account for cerebrospinal fluid contamination, partial volume effects could still introduce bias in estimates within mixed tissue voxels (Zhang et al. 2012). Alternative techniques, such as constrained NODDI (C‐NODDI; Alsameen et al. 2023) or multi‐TE NODDI (Gong et al. 2020), could help improve the interpretability of NODDI‐based findings in future neuroimaging research. WMTI, by extending DKI through the separation of intra‐ and extra‐axonal contributions (Fieremans et al. 2010, 2011), relies on strong biophysical assumptions, such as the absence of water exchange between compartments, assumptions that are often violated in regions with fiber crossings or complex geometries.

Relaxometry and MTI offer valuable insights into tissue composition, particularly regarding myelin content, but also present interpretative challenges. Although relaxometry offers direct quantification of relaxation times that reflect tissue microstructure, these measurements are influenced by multiple biological factors, such as water content, myelin, and iron, thereby complicating the isolation of specific contributors (Deoni 2010). MTI metrics, such as MTR, are sensitive to macromolecular content but lack specificity to myelin and can be affected by inflammation, edema, or axonal loss (Sled 2018). Furthermore, MTR values are highly dependent on acquisition parameters (Sled 2018), which complicate cross‐study comparisons. Multicomponent relaxometry, including multi‐echo spin‐echo T_2_, mcDESPOT, and BMC‐mcDESPOT (Bouhrara and Spencer 2017; Deoni et al. 2008; MacKay et al. 2006), and quantitative MTI approaches (Sled 2018), offer improved specificity to myelin. Consequently, future studies employing these advanced techniques would be informative to better understand the role of myelin in autism.

## 7 Limitations

There are several limitations to consider when interpreting the findings of this review. First, a comprehensive backward and forward citation search was not conducted, which may have excluded relevant studies and limited the scope of the review. Second, many studies did not consistently report relevant variables. In some cases, findings such as reduced MWF or higher FISO in autism were based on a single study, which undermines the robustness of the conclusions. Third, while group differences and associations were synthesized, many studies reported non‐significant findings, introducing variability to the overall conclusions. Diverse analytical methods (e.g., tract‐based or GM‐based spatial statistics) may have also resulted in inconsistent outcomes. Fourth, synthesizing findings by regions across techniques necessitated broad groupings (e.g., combining the corpus callosum with subregions such as the splenium), which might obscure sub‐regional specificity. Finally, including participants across a wide age range from early childhood to adulthood captured developmental trends; however, age‐specific impacts were not assessed, limiting insights into how these imaging markers vary across developmental stages. This also highlights a significant gap in research on neurodegeneration in aging autistic individuals, as most studies have focused on specific developmental stages (childhood, adolescence, or adulthood), with less emphasis on neurobiological changes related to aging.

## 8 Implications

Variability in acquisition parameters across studies highlights the need for more standardized protocols. While complete standardization may not be feasible due to diverse research goals, resources, and populations, flexible guidelines and transparent reporting could enhance reproducibility. Additionally, adopting open science practices, such as data sharing, would enable consistent processing pipelines and harmonization techniques, facilitating better cross‐study comparisons. Consistent group differences in NDI, FD, and FDC provide a foundation for further exploration of neurobiological mechanisms in autism. However, variability in DKI, WMTI, and relaxometry metrics suggests that identifying universal biomarkers may be challenging. Future research should emphasize subgroup analyses to address heterogeneity in autism, considering how differences in age, symptom profiles, and developmental trajectories might influence brain microstructure. Incorporating diverse control groups will also be essential for distinguishing autism‐specific changes from those in other developmental conditions. Moreover, longitudinal studies are vital for tracking how imaging markers evolve and relate to developmental milestones. While cross‐sectional studies are informative and provide snapshots of neurodevelopmental differences, longitudinal research could identify biomarkers predictive of developmental outcomes and guide targeted interventions. This approach would also clarify the progression of brain plasticity and microstructural changes across life stages. In conclusion, current findings underscore the need for greater standardization, validation, and longitudinal research in advanced quantitative microstructure imaging in autism research. Subgroup analyses, replication studies, and targeted support strategies addressing WM integrity represent important next steps for advancing the field.

## Author Contributions

**C.D.Y.:** conceptualized the review, reviewed the literature, extracted data, drafted the manuscript, and revised and approved the final version. **D.C.D.:** conceptualized the review, verified the included studies and the extracted data, and revised and approved the final version.

## Ethics Statement

The authors have nothing to report.

## Conflicts of Interest

The authors declare no conflicts of interest.

## Supporting information


**Table S1:** Biological features measured by the parameters of each advanced quantitative MRI.

## Data Availability

Data sharing is not applicable to this article.
