# Supplementary material for: Advanced Quantitative Microstructure Imaging in Autism: A Review of Methodology, Group Differences, and Associations With Developmental Outcomes
Source: Autism Res. 2025 Oct 6;18(11):2140–65. doi: 10.1002/aur.70122 (PMC12661291; doi:10.1002/aur.70122)
Supplement: Supplementary file 1 — Table S1: Biological features measured by the parameters of each advanced quantitative MRI. [file AUR-18-2140-s001.docx]

**SUPPLEMENTARY TABLE 1.** Biological features measured by the parameters of each advanced quantitative MRI.

| Metric | Higher Value | Lower Value |
| --- | --- | --- |
| Diffusion MRI Signal Representations | | |
| Diffusion Kurtosis Imaging (DKI) | | |
| Mean kurtosis (MK) | Greater tissue complexity or diffusion barriers | Reduced tissue complexity, possibly degeneration or simplification |
| Axial kurtosis (AK) | More restriction along axons, possibly indicating intact axonal structure | Reduced axonal integrity or axonal injury |
| Radial kurtosis (RK) | More restriction perpendicular to axons, possibly indicating intact myelin or radial | Reduced myelination or extracellular expansion |
| Fractional anisotropy of kurtosis (FAK) | Greater microstructural complexity, directional heterogeneity, or tissue organization (e.g., intact fiber bundles) | Reduced microstructural complexity, less directional dependence of diffusion, or tissue disruption (e.g., degeneration, edema) |
| Constrained Spherical Deconvolution (CSD) | | |
| Fiber density (FD) | Increased intra-axonal content along a fiber orientation | Reduced axonal density or fiber content |
| Fiber cross-section (FC) | Larger tract or bundle size (macrostructure) | Smaller tract size or atrophy |
| Product of FD and FC (FDC) | Greater overall fiber content (combined micro- and macrostructure) | Reduced fiber content (due to micro- or macrostructural loss) |
| Diffusion MRI Biophysical Models | | |
| Composite Hindered and Restricted Model of Diffusion (CHARMED) | | |
| Restricted | Greater axonal density or intra-axonal volume | Lower axonal density or disrupted intra-axonal space |
| Hindered | Less extracellular restriction, possibly indicating axonal loss or demyelination | More extracellular restriction, possibly due to denser cellular packing |
| Neurite Orientation Dispersion and Density Imaging (NODDI) | | |
| Neurite density index (NDI) | Higher neurite or axon density/packing | Reduced neurite density, possibly degeneration or pruning |
| Orientation dispersion index (ODI) | Greater angular dispersion of neurite orientations (e.g., cortex) | Less dispersed, more coherent fiber orientations (e.g., white matter tracts) |
| Isotropic volume fraction (FISO) | Increased free water, cerebrospinal fluid, or extracellular content (e.g., due to atrophy or inflammation) | Less free water, more compact or healthy tissue |
| White Matter Tract Integrity (WMTI) | | |
| Axonal water fraction (*f_axon_*) | Greater axonal density/packing | Reduced axonal density/packing, possible edema or extracellular expansion |
| Intra-axonal diffusivity (*D_axon_*) | More preserved intra-axonal structure or axonal swelling | More cytoskeletal disruption or intra-axonal debris |
| Extra-axonal axial diffusivity (*AD_extra_*) | More loss of axonal packing or extra-axonal barriers | Greater gliosis or extracellular crowding |
| Extra-axonal radial diffusivity (*RD_extra_*) | More myelin breakdown or demyelination | Greater myelin preservation or myelin density |
| Extra-axonal tortuosity | Greater axonal organization | Reduced axonal organization |
| Relaxometry / Multicomponent Relaxometry | | |
| Longitudinal relaxation time (*T_1_*) | More free water, or less mature or organized tissue | Higher macromolecular or myelin content, or more organized tissue |
| Transverse relaxation time (*T_2_*) | More mobile water (e.g., edema, inflammation), or reduced tissue organization | Greater tissue density, myelin, or substances that cause faster signal loss (e.g., iron) |
| *T_2_’* | Reduced values in presence of iron or vascular pathology (susceptibility effects) | Less iron or vascular abnormalities (less susceptibility-related signal loss) |
| Myelin water fraction (MWF) | Greater myelin content | Lower myelin content |
| Magnetization Transfer Imaging (MTI) | | |
| Magnetization transfer ratio (MTR) | Greater macromolecular content, often myelin | Reduced macromolecular content, possibly demyelination |
